# Supplementary material for: Widespread Detection of Fowl Adenovirus Serotype 2/11 Species D Among Cases of Inclusion Body Hepatitis–Hydropericardium Syndrome in Chickens in Egypt
Source: Microorganisms. 2025 May 12;13(5):1107. doi: 10.3390/microorganisms13051107 (PMC12114069; doi:10.3390/microorganisms13051107)
Supplement: Supplementary file 1 [file microorganisms-13-01107-s001.zip › microorganisms-3607538-supplementary.pdf]

Supplementary Table S1. Available data of fowl adenovirus-positive flocks detected from different investigated provinces.

Province	Sample No.	Sample code	Age	Date of sample collection	No. of birds in the flock	system of housing	Clinical signs	Gross lesions	Mortality percent
Beni-Suef	1	1 SB	5 days	20-10-2020	3500	Deep litter	unavailable	Hepatitis, hydropericardium	unavailable
	2	2 SB	35 days	29-10-2020	800	Deep litter	unavailable	Hepatitis, hydropericardium	unavailable
	3	3 SB	23 days	13-2-2021	3000	Deep litter	unavailable	Hepatitis, hydropericardium	unavailable
	4	4 SB	30 days	4/1/2021	4000	Deep litter	unavailable	Hepatitis, hydropericardium	unavailable
	5	5 SB	33 days	8/2/2021	6000	Deep litter	unavailable	Hepatitis, hydropericardium	unavailable
	6	6 SB	19 days	10/2/2021	8000	cages	unavailable	Hepatitis, hydropericardium	unavailable
	7	7 SB	20 days	13-2-2021	4000	Deep litter	unavailable	Hepatitis, hydropericardium	unavailable
	8	8 SB	32 days	25-2-2021	700	Deep litter	unavailable	Hepatitis hydropericardium	unavailable
	9	10 SB	25 days	16-2-2022	6000	Deep litter	unavailable	Hepatitis hydropericardium	unavailable
	10	11 SB	29 days	16-2-2022	11000	Deep litter	unavailable	Hepatitis	unavailable
	11	12 SB	25 days	5/3/2022	1500	Deep litter	unavailable	hydropericardium Liver cirrhosis	unavailable
	12	13 SB	31 days	28-3-2022	2600	Deep litter	unavailable	hydropericardium Hepatitis	unavailable
	13	HB	41 days	28-12-2021	600	cages	Resp.signs Diarrhea High MR%	Hepatitis hydropericardium CRD	6%
	14	15 SB	27 days	27-6-2022	7000	cages	Stunting 300 of 7000	CRD Proventriculitis	Normal MR
	15	24 SB	30 days	29-6-2022	4500	Deep litter	Stunting 2.2%	CRD Ascites hydropericardium	Normal MR
	16	28 SB	19 days	30-6-2022	11800	cages	unavailable	CRD, ascites Peritonitis Flaccidity of proventriculus Necrotic enteritis	Normal MR
	17	38 SB	21 days	19-7-2022	40000	Deep litter	Normal B.WT Normal FCR	CRD Congested liver	Normal MR

								Pneumonia<br>Necrotic enteritis<br>Hepatic rupture	
	18	ODO 1	One day	28-3-2022		Deep litter		Non-specific	
	19	ODO 2	One day	28-3-2022		Deep litter		Non-specific	
	20	ODO 3	One day	17-5-2022		Deep litter		Non-specific	
Fayoum	21	1 MF	10 days	8/2/2021	11800	Deep litter	High MR<br>Resp. signs<br>Diarrhea	hydropericardium, Hepatitis , Ascites<br>Liver cirrhosis<br>Nephritis<br>CRD<br>Stunting<br>Caseous plug, pneumonia	2.5 % in the first 10 days.
	22	2 MF	31 days	8/2/2021	12000	cages	Depression<br>High MR<br>gasping	hydropericardium, Hepatitis<br>CRD<br>Severe tracheitis<br>Pericarditis	0.8 % within 3 days.
	23	3 MF	19 days	7/3/2021	5500	Deep litter	Bloody diarrhea	E.Tennella<br>Necrotic enteritis<br>CRD<br>Liver cirrhosis<br>Ascites<br>Congested kidney<br>Pneumonia	Normal
	24	5 MF	32 days	7/3/2021	8000	cages	High MR<br>White diarrhea	hydropericardium, hepatitis, CAS, CRD<br>Severe tracheitis & bronchitis<br>Congested liver<br>Pneumonia<br>Blue wing<br>Enteritis	2.5 % within 2 days.
	25	6 MF	26 days	7/3/2021	3000	Deep litter	Poor FCR<br>White diarrhea	hydropericardium Liver cirrhosis<br>CRD<br>Pneumonia<br>Enteritis	Normal MR
	26	7 MF	28 days	7/3/2021	5000	cages	unavailable	hydropericardium, Hepatitis<br>Liver cirrhosis	Unknown

							CRD, CAS	
27	8 MF	35 days	12/6/2021	5500	Deep litter	Poor feed consumption	hydropericardium, hepatitis<br>Liver cirrhosis<br>Caseous plug<br>Pale liver<br>Nephritis<br>Pneumonia<br>Tracheitis	1.6% MR within 3 days.
28	9 MF	28 days	11/4/2021	4500	Deep litter	Poor feed consumption	hydropericardium, hepatitis<br>Stunting<br>Nephritis<br>Caseous plug<br>Liver cirrhosis	0.8% within 2 days.
29	10 MF	32 days	4-4-2021	20000	Deep litter	unavailable	hepatitis, hydropericardium	unavailable
30	11 MF	14 days	19-9-2021	10000	Deep litter	Depression	Liver cirrhosis<br>Hepatitis<br>Enlarged liver<br>CAS<br>Enteritis	0.6% within 3 days.
31	12 MF	30 days	19-9-2021	5000	Deep litter	Undigested food	hepatitis, pneumonia<br>Pale intestine<br>Nephritis<br>CRD	unavailable
32	13 MF	37 days	8/4/2021	12000	Deep litter	Gasping<br>Depression<br>High mortalities	hydropericardium, Liver necrosis<br>Enlarged liver.<br>CRD<br>Caseous plug<br>Severe tracheitis<br>Pneumonia	3.7 % within 3 days.
33	18 MF	27 days	26-2-2022	8000	Deep litter	Depression	hepatitis, hydropericardium	0.45 % within 2 days.
34	19 MF	22 days	26-2-2022	7000	cages	High MR	hepatitis Pneumonia<br>CRD, AS<br>Ulcers on payer’s patches	2 % within 2 days.
35	20 MF	10 days	21-2-2022	5000	Deep litter	Depression	hepatitis, Liver cirrhosis<br>Caseous plug<br>CRD	Normal
36	22 MF	33 days	27-2-2022	12000	cages	unavailable	hepatitis Pneumonia<br>CRD	0.5 % within days.

							Head cyanosis<br>Tracheitis<br>Submandibular edema	
37	23 MF	35 days	28-2-2022	3000	cages	Undigested food	hepatitis, Ascites	unavailable
38	25a MF	31 days	28-2-2022	10500	Deep litter	Unilateral Lameness<br>High MR<br>Depression	hepatitis, AS<br>CRD<br>Caseated bursa<br>Nephrosis	0.5 % within 2 days.
39	25b MF	26 days	28-2-2022	3000	Deep litter	High MR<br>Gasping<br>Depression<br>Prostration<br>Death	Hepatitis, CRD<br>Liver cirrhosis<br>Peritonitis<br>Ulcer on cecal tonsils<br>Caseated bursa<br>Internal hemorrhage	6 % within 3 days.
40	32 MF	35 days	6-3-2022	14000	Deep litter	unavailable	hepatitis,<br>ascites, head cyanosis<br>CRD, Pneumonia	unavailable
41	39 MF	31 days	21-2-2022	4600	Deep litter	unavailable	hepatitis	unavailable
42	40 MF	28 days	1/3/2022	3600	cages	White diarrhea<br>Depression<br>Ascites	hepatitis, CRD<br>Liver cirrhosis<br>Pericarditis<br>Tracheitis	2 % within 2 days.
43	41 MF	32 days	20-3-2022	5500	cages	Poor FCR	hepatitis, CRD<br>Necrotic enteritis<br>Pneumonia<br>Ascites<br>Enteritis	Normal
44	42 MF	33 days	26-3-2022	17500	Deep litter	High MR	hepatitis, CRD<br>Pneumonia,<br>Tracheitis,<br>Caseated bursa<br>Nephritis	4.2 % within 3 days
45	44 MF	35 days	24-4-2022	4000	cages	High MR<br>Lameness	hepatitis, HPS CRD<br>Pneumonia<br>Necrotic enteritis<br>General congestion	High

46	45 MF	32 days	24-4-2022	11500	Deep litter	unavailable	hepatitis, Pneumonia CAS, CRD, necrotic enteritis	14 chicks per day.
47	46 MF	32 days	8/6/2022	4000	Deep litter	High MR	CRD	unavailable
48	47 MF	34 days	30-6-2022	32000	cages	High MR	hepatitis, tracheitis CRD, pneumonia ulcers on the cecal tonsils.	0.7 % within 2 days.
49	48 MF	23d	28-6-2022	7000	cages	Depression, Lameness, rales diarrhea	CRD hepatitis, ulcers on cecal tonsils nephritis	1.7 within 3 days.
50	54 MF	17 days	19/7/2022	5000	Deep litter	High MR low viability Depression un-uniformity B.wt	CRD, pneumonia Retained yolk sac. Enteritis	9 % within 3 days.
51	60 MF	11 days	26-7-2022	5000	Deep litter	Vaccinated flock Normal flock	Pale intestine Proventriculitis	Normal MR
52	61 MF	11 days	26-7-2022	4500	Deep litter	Normal FCR Coughing	Normal picture	Normal MR
53	62 MF	22 days	26-7-2022	5500	cages	Normal MR	Enteritis Swollen head Necrotic enteritis Pale intestine	Normal MR
54	63 MF	22 days	26-7-2022	9000	cages	Normal MR Normal FCR	Partial caseous plug NS	Normal MR
55	66 MF	20 days	16-8-2022	7000	cages	Normal MR Within the first 4 days	Chronic CRD Stunting Enteritis	Normal MR
56	67 MF	33 days	16-8-2022	4000	Deep litter	White diarrhea Gasping	Pneumonia CRD, severe tracheitis Caseous plug	1% within 3 days.
57	68 MF	21 days	16-8-2022	3000	Deep litter	Normal MR swollen head	CRD, pneumonia necrotic enteritis	normal MR in the first two days.
58	9 SF	35 days	3-3-2021	7800	Deep litter	off food poor FCR rales	CRD, pneumonia Enteritis	1.6 within 2 days.

	59	10 SF	28 days	3-3-2021	10000	Deep litter	bloody diarrhea	hepatitis	normal MR
Giza	60	I MG	44 days	16-5-2022	6000	Deep litter	gasping<br>swollen head<br>lameness<br>stunting	hydropericardium, CRD,<br>pericarditis<br>pneumonia<br>AS, tracheitis	unavailable
	61	3 MG	40 days	16-5-2022	9200	Deep litter	unavailable	hydropericardium, hepatitis<br>CRD, pneumonia<br>pericarditis<br>caseous bronchitis	14 % within 11 days.
	62	15 MG	33 days	15-8-2022	28278	Deep litter	unavailable	Hepatitis, hydro and hemopericardium CRD,<br>ascites<br>liver cirrhosis<br>peritonitis	high MR within the first days of life then return to normal<br>35 chicks per day at 33 days of age.
	63	28 MG	22 days	15-8-2022	4700	Deep litter	unavailable	N.S	normal MR
Behira	64	2 MG	35 days	16-5-2022	13500	Deep litter	high MR<br>brownish diarrhea<br>swollen head<br>lameness	Hepatitis, enlarged liver	1 % within one day
Menia	65	9 SB	28 days	27-2-2021	5000	Deep litter	Normal MR	Hepatitis	Unavailable
	66	16 MF	33 days	20-2-2022	14000	Deep litter	Poor FCR<br>gasping<br>white diarrhea<br>un-uniformity B.wt	Hepatitis, CAS, peritonitis,<br>severe tracheitis<br>pneumonia<br>ulcer on the cecal tonsils.	0.5 % MR within 3 days.

Supplementary Figure S1. Nucleotide sequence (A) and deduced amino acid sequence (B) of strains FAdV serotype 2/11 species D included in the current study

A. Nucleotide sequence analysis

```
#MT975968   GTAGTCGCGC CCACTCGCAA TGTCACCACC GAGAAGGCTC AGAGGCTTCA GATCAGGTTT TACCCGACGC AGACCGACGA CACGCCCAAC AGTTACCGCG TGCGGTACAG TTTAAACGTG GGCGACAGTT GGGTTCTTGA CATGGGAGCC [ 309]
#KT862805   .......... .......... .......... .......... .......... .......... .......... .......... .......... .......... .......... .......... .......... .......... ......C...
#KX247375   ---------- ---------- ---------- ---------- ---------- ---------- ---------- ---------- ---------- ---------- ---------- ---------- ---------- ---------- ----------
#PP993159   ---------- ---------- ---------- ---------- ---------- ---------- ---------- ---------- ---------- ------.... .......... .......... ..T....... .......... ......C...
#PP993160   ---------- --------.. .......... .......... .......... .......... .......... .......... .......... .......... .......... .......... ..T....... .......... ......C...
#PP993161   ---------- ---------- ----...... .......... .......... .......... .......... .......... .......... .......... .......... .......... ..T....... .......... ......C...
#PP993162   ---------- ---------- ---------- ---------- ---------- ---------- ---------- ---------- ---------- ------.... .......... .......... ..T....... .......... ......C...
#PP993163   ---------- ---------- -......... .......... .......... .......... .......... .......... .......... .......... .......... .......... ..T....... .......... ......C...
#PP993164   ---------- ---------- -------... .......... .......... .......... .......... .......... .......... .......... .......... .......... ..T....... .......... ......C...
#PP993165   ---------- ---------- ---------- ---------- ---------- ---------- ---------- --........ .......... G......... .......... .......... ..T....... .......... ......C...
#PP993166   ---------- ---------- ----...... .......... .......... .......... .......... .......... .......... .......... .......... .......... ..T....... .......... ......C...
#PP993167   ---------- ---------- -------... .......... .......... .......... .......... .......... .......... .......... .......... .......... ..T....... .......... ......C...
#PP993168   ---------- ---------- -------... .......... .......... .......... .......... .......... .......... .......... .......... .......... ..T....... .......... ......C...
#PP993169   ---------- ---------- -------... .......... .......... .......... .......... .......... .......... .......... .......... .......... ..T....... .......... ......C...
#PP993170   ---------- ---------- -------... .......... .......... .......... .......... .......... .......... .......... .......... .......... ..T....... .......... ......C...
#PP993171   ---------- ---------- ---------- ---------. .......... .......... .......... .......... .......... .......... .......... .......... ..T....... .......... ......C...
#PP993172   ---------- ---------- ----T..... .......... .......... .......... .......... .......... .......... .......... .......... .......... ..T....... .......... ......C...
#PP993173   ---------- ---------- ---------- ------.... .......... .......... .......... .......... .......... .......... .......... .......... ..T....... .......... ......C...
#PP993174   ---------- ---------- ---------- ------.... .......... .......... .......... .......... .......... .......... .......... .......... ..T....... .......... ......C...
#PP993175   ---------- ---------- -......... .......... .......... .......... .......... .......... .......... .......... .......... .......... ..T....... .......... ......C...
#PP993176   ---------- ---------- ---------- ------.... .......... .......... .......... .......... .......... .......... .......... .......... ..T....... .......... ......C...
#PP993177   ---------- ---------- ---------- ------.... .......... .......... .......... .......... .......... .......... .......... .......... ..T....... .......... ......C...

#MT975968   ACCTACTTCG ACATCAAGGG CGTCCTAGAC AGAGGACCTT CTTTTAAACC GTATGGAGGA ACCGCATACA ATCCCCTCGC GCCCCGCGAA GCCTTTTTCA ACAATTGGGT TGACACAGAG GCGAGCAAGA CCGTCATCAC GGGTCAGATG [ 459]
#KT862805   .......... .......... .......... .......... .......... A..C..G..T ..G..T.... .......... T..T...... ..G....... .......A. C.O.T..C.G TA.C.A.... .A........ ..........
#KX247375   ---------- ---------- ---------- ---------- --------.. .......... .......... .......... .......... .......... .......... .......... .......... .......... ..........
#PP993159   .......... .......... .......... .......... .......... ...C..G..C ..G..T.... .......... T..T...... ..G....... .......A. C..T..C.GT A.C.A..... .A........ ..........
#PP993160   .......... .......... .......... .......... .......... ...C..G..C ..G..T.... .......... T..T...... ..G....... .......A. C..T..C.GT A.C.A..... .A........ ..........
#PP993161   .......... .......... .......... .......... .......... ...C..G..C ..G..T.... .......... T..T...... ..G....... .......A. C..T..C.GT A.C.A..... .A........ ..........
#PP993162   .......... .......... .......... .......... .......... ...C..G..C ..G..T.... .......... T..T...... ..G....... .......A. C..T..C.GT A.C.A..... .A........ ..........
#PP993163   .......... .......... .......... .......... .......... ...C..G..C ..G..T.... .......... T..T...... ..G....... .......A. C..T..C.GT A.C.A..... .A........ ..........
#PP993164   .......... .......... .......... .......... .......... ...C..G..C ..G..T.... .......... T..T...... ..G....... .......A. C..T..C.GT A.C.A..... .A........ ..........
#PP993165   .......... .......... .......... .......... .......... ...C..G..C ..G..T.... .......... T..T...... ..G....... .......A. C..T..C.GT A.C.A..... .A........ ..........
#PP993166   .......... .......... .......... .......... .......... ...C..G..C ..G..T.... .......... T..T...... ..G....... .......A. C..T..C.GT A.C.A..... .A........ ..........
#PP993167   .......... .......... .......... .......... .......... ...C..G..C ..G..T.... .......... T..T...... ..G....... .......A. C..T..C.GT A.C.A..... .A........ ..........
#PP993168   .......... .......... .......... .......... .......... ...C..G..C ..G..T.... .......... T..T...... ..G....... .......A. C..T..C.GT A.C.A..... .A........ ..........
#PP993169   .......... .......... .......... .......... .......... ...C..G..C ..G..T.... .......... T..T...... ..G....... .......A. C..T..C.GT A.C.A..... .A........ ..........
#PP993170   .......... .......... .......... .......... .......... ...C..G..C ..G..T.... .......... T..T...... ..G....... .......A. C..T..C.GT A.C.A..... .A........ ..........
#PP993171   .......... .......... .......... .......... .......... ...C..G..C ..G..T.... .......... T..T...... ..G....... .......A. C..T..C.GT A.C.A..... .A........ ..........
#PP993172   .......... .......... .......... .......... .......... ...C..G..C ..G..T.... .......... T..T...... ..G....... .......A. C..T..C.GT A.C.A..... .A........ ..........
#PP993173   .......... .......... .......... .......... .......... ...C..G..C ..G..T.... .......... T..T...... ..G....... .......A. C..T..C.GT A.C.A..... .A........ ..........
#PP993174   .......... .......... .......... .......... .......... ...C..G..C ..G..T.... .......... T..T...... ..G....... .......A. C..T..C.GT A.C.A..... .A........ ..........
#PP993175   .......... .......... .......... .......... .......... ...C..G..C ..G..T.... .......... T..T...... ..G....... .......A. C..T..C.GT A.C.A..... .A........ ..........
#PP993176   .......... .......... .......... .......... .......... ...C..G..C ..G..T.... .......... T..T...... ..G....... .......A. C..T..C.GT A.C.A..... .A........ ..........
#PP993177   .......... .......... .......... .......... .......... ...C..G..C ..G..T.... .......... T..T...... ..G....... .......A. C..T..C.GT A.C.A..... .A........ ..........

#MT975968   ACAACTCCCT ACGAAAACGT CCAGGGCGCT AAAGACAAGA CTGCCGCGAT CGTCGCCGCT CTTTCAGGGG TTTATCCCGA TCCCAATATC GGTACCGCCA TCAGCGAGAT GGGCGCCTTA GACGCGACGT CGGCAGCCCA AGTCGGATTG [ 609]
#KT862805   .......... .........A ..G.A..... .......... .......... .......... .......... .......... .......... ..CC...... .......... .......... ....A..... ......A... ..........
#KX247375   .......... .......... .......... .......... .......... .......... .......... .......... .......... .......... .......... .......... A......... .......... ..........
#PP993159   .......... .T........ ....A..... .......... .......... .......... .......... .......... .......... .......... .......... .......... A..CA..... ......AG.........................A
#PP993160   .......... .T........ ....A..... .......... .......... .......... .......... .......... .......... .......... .......... .......... A..CA..... ......AG.........................A
```

```
#PP993161    .......... .T........ ...A...... .......... .......... .......... .......... .......... .......... .......... .......... .......... ..........A..CA..... .....AG.........................A
#PP993162    .......... .T........ ...A...... .......... .......... .......... .......... .......... .......... .......... .......... .......... ..........A..CA..... .....AG.........................A
#PP993163    .......... .T........ ...A...... .......... .......... .......... .......... .......... .......... .......... .......... .......... .......... ...CA..... .....AG.........................A
#PP993164    .......... .T........ ...A...... .......... .......... .......... .......... .......... .......... .......... .......... .......... .......... ...CA..... .....AG.........................A
#PP993165    .......... .T........ ...A...... ......C... .......... .......... .......... .......... .......... .......... .......... .......... .......... ...CA..... .....AG.........................A
#PP993166    .......... .T........ ...A...... .......... .......... .......... .......... .......... .......... .......... .......... .......... ..........A..CA..... .....AG.........................A
#PP993167    .......... .T........ ...A...... .......... .......... .......... .......... .......... .......... .......... .......... .......... .......... ...CA..... .....AG.........................A
#PP993168    .......... .T........ ...A...... .......... .......... .......... .......... .......... .......... .......... .......... .......... .......... ...CA..... .....AG.........................A
#PP993169    .......... .T........ ...A...... .......... .......... .......... .......... .......... .......... .......... .......... .......... ..........A..CA..... .....AG.........................A
#PP993170    .......... .T........ ...A...... .......... .......... .......... .......... .......... .......... .......... .......... .......... ..........A..CA..... .....AG.........................A
#PP993171    .......... .T........ ...A...... .......... .......... .......... .......... .......... .......... .......... .......... .......... ..........A..CA..... .....AG.........................A
#PP993172    .......... .T........ ...A...... .......... .......... .......... .......... .......... .......... .......... .......... .......... ..........A..CA..... .....AG.........................A
#PP993173    .......... .T........ ...A...... .......... .......... .......... .......... .......... .......... .......... .......... .......... ..........A..CA..... .....AG.........................A
#PP993174    .......... .T........ ...A...... .......... .......... .......... .......... .......... .......... .......... .......... .......... ..........A..CA..... .....AG.........................A
#PP993175    .......... .T........ ...A...... .......... .......... .......... .......... .......... .......... .......... .......... .......... ..........A..CA..... .....AG.........................A
#PP993176    .......... .T........ ...A...... .......... .......... .......... .......... .......... .......... .......... .......... .......... ..........A..CA..... .....AG.........................A
#PP993177    .......... .T........ ...A...... .......... .......... .......... .......... .......... .......... .......... .......... .......... .......... ...CA..... .....AG.........................A

#MT975968    GCTGCCCGAT TCGCGAAAGT GTCGAGCGAT AACACGCGTC TAGCCTACGG AGCCTACGTT AAACCGCTCA AGAACGACGG TTCTCAATCG ATTAACCCCA CTCCTTACTG GGTCATGGTA AGCAACGCCA CAAACTATCT CGGAGTCATG [ 759]
#KT862805    .......... .......... .......... .......... .......... .......... .......... .......... .......... .......... .......... ........AC ....GT...G ..G.A..... ..........
#KX247375    .......... .......... A......... .......... .......... .......... .......... .......... .......... .......... .......... ........AC .......... .......... ..........
#PP993159    .......... .......... .......... ........A. .......... .......... .......... .......... .......... .......... .......... ........AC .....T.... ..G.A..... ..........
#PP993160    .......... .......... .......... ........A. .......... .......... .......... .......... .......... .......... .......... ........AC .....T.... ..G.A..... ..........
#PP993161    .......... .......... .......... ........A. .......... .......... .......... .......... .......... .......... .......... ........AC .....T.... ..G.A..... ..........
#PP993162    .......... .......... .......... ........A. .......... .......... .......... .......... .......... .......... .......... ........AC .....T.... ..G.A..... ..........
#PP993163    .......... .......... .......... ........A. .......... .......... .......... .......... .......... .......... .......... ........AC .....T.... ..G.A..... ..........
#PP993164    .......... .......... .......... ........A. .......... .......... .......... .......... .......... .......... .......... ........AC .....T.... ..G.A..... ..........
#PP993165    .......... .......... .......... ........A. .......... .......... .......... .......... .......... .......... .......... ........AC .....T.... ..G.A..... ..........
#PP993166    .......... .......... .......... ........A. .......... .......... .......... .......... .......... .......... .......... ........AC .....T.... ..G.A..... ..........
#PP993167    .......... .......... .......... ........A. .......... .......... .......... .......... .......... .......... .......... ........AC .....T.... ..G.A..... ..........
#PP993168    .......... .......... .......... ........A. .......... .......... .......... .......... .......... .......... .......... ........AC .....T.... ..G.A..... ..........
#PP993169    .......... .......... .......... ........A. .......... .......... .......... .......... .......... .......... .......... ........AC .....T.... ..G.A..... ..........
#PP993170    .......... .......... .......... ........A. .......... .......... .......... .......... .......... .......... .......... ........AC .....T.... ..G.A..... ..........
#PP993171    .......... .......... .......... ........A. .......... .......... .......... .......... .......... .......... .......... ........AC .....T.... ..G.A..... ..........
#PP993172    .......... .......... .......... ........A. .......... .......... .......... .......... .......... .......... .......... ........AC .....T.... ..G.A..... ..........
#PP993173    .......... .......... .......... ........A. .......... .......... .......... .......... .......... .......... .......... ........AC .....T.... ..G.A..... ..........
#PP993174    .......... .......... .......... ........A. .......... .......... .......... .......... .......... .......... .......... ........AC .....T.... ..G.A..... ..........
#PP993175    .......... .......... .......... ........A. .......... .......... .......... .......... .......... .......... .......... ........AC .....T.... ..G.A..... ..........
#PP993176    .......... .......... .......... ........A. .......... .......... .......... .......... .......... .......... .......... ........AC .....T.... ..G.A..... ..........
#PP993177    .......... .......... .......... ........A. .......... .......... .......... .......... .......... .......... .......... ........AC .....T.... ..G.A..... ..........

#MT975968    GGAGTCGAAG ACTTTAGCGC CTCGCTAACC TATCCCGATA CGCTCCTCAT TCCCCCGCCG ACCGAATACT CAGAAGTGAA TAGGCCCGTC ATGAAGGCAA ACAGGCCGAA TTACATCGGA TTTAGGGACA ATTTTATCAA CCTGCTCTAT [ 909]
#KT862805    .......... .......... .......... ........C. .......T.. .......... ........T. .......... ..CCGG.... .......... .......... .......... .......... .......... ..........
#KX247375    .......... .......... .......... .......... .......T.. .........A .......... .......... ..CCGG.... .......... .......... .......... .......... .......... ..........
#PP993159    .......... .......... T......... ........C. .......T.. .......... .......... .......... ..CCGG.... .......... .......... .......... .......... .......... ..........
#PP993160    .......... .......... T......... ........C. .......T.. .......... .......... .......... ..CCGG.... .......... .......... .......... .......... .......... ..........
#PP993161    .......... .......... T......... ........C. .......T.. .......... .......... .......... ..CCGG.... .......... .......... .......... .......... .......... ..........
#PP993162    .......... .......... T......... ........C. .......T.. .......... .......... .......... ..CCGG.... .......... .......... ......T... .......... .......... ..........
#PP993163    .......... .......... T......... ........C. .......T.. .......... .......... .......... ..CCGG.... .......... .......... .......... .......... .......... ..........
#PP993164    .......... .......... T......... ........C. .......T.. .......... .......... .......... ..CCGG.... .......... .......... .......... .......... .......... ..........
#PP993165    .......... .......... T......... ........C. .......T.. .......... .......... .......... ..CCGG.... .......... .......... .......... .......... .......... ..........
#PP993166    .......... .......... T......... ........C. .......T.. .......... .......... .......... ..CCGG.... .......... .......... .......... .......... .......... ..........
#PP993167    .......... .......... T......... ........C. .......T.. .......... .......... .......... ..CCGG.... .......... .......... .......... .......... .......... ..........
#PP993168    .......... .......... T......... ........C. .......T.. .......... .......... .......... ..CCGG.... .......... .......... .......... .......... .......... ..........
#PP993169    .......... .......... T......... ........C. .......T.. .......... .......... .......... ..CCGG.... .......... .......... .......... .......... .......... ..........
#PP993170    .......... .......... T......... ........C. .......T.. .......... .......... .......... ..CCGG.... .......... .......... .......... .......... .......... ..........
#PP993171    .......... .......... T......... ........C. .......T.. .......... .......... .......... ..CCGG.... .......... .......... .......... .......... .......... ..........
#PP993172    .......... .......... T......... ........C. .......T.. .......... .......... .......... ..CCGG.... .......... .......... .......... .......... .......... ..........
#PP993173    .......... .......... T......... ........C. .......T.. .......... .......... .......... ..CCGG.... .......... .......... .......... .......... .......... ..........
#PP993174    .......... .......... T......... ........C. .......T.. .......... .......... .......... ..CCGG.... .......... .......... .......... .......... .......... ..........
#PP993175    .......... .......... T......... ........C. .......T.. .......... .......... .......... ..CCGG.... .......... .......... .......... .......... .......... ..........
```

```
#PP993176    .......... .......... T......... ........C. .......T-- ---------- ---------- ---------- --CCGG.... .......... .......... .......... .......... .......... ..........
#PP993177    .......... .......... T......... ........C. .......T-- ---------- ---------- ---------- --CCGG.... .......... .......... .......... .......... .......... ..........

#MT975968    CATGATACGG GTGTGTGCTC GGGTACTCTG AATTCGGAGC GTTCGGGTAT GAACGTCGTC GTGGAGCTCC AGGACAGAAA CACGGAACTC AGTTACCAGT ACATGTTAGC CGATATGATG TCGCGGCATC ACTACTACGC GCTTTGGAAC [ 1059]
#KT862805    .......... .......... .......... .......... .......... .......... ..C....... .......... .......... .......... .......... .......... .......... ....T..T.. ..........
#KX247375    .......... .......... .......... .......... .......... .......... ..C....... .......... .........T .......... .......... .......... .......... ....T..... ..........
#PP993159    .......... .......... A......... .......... .A........ .......... ..C....... .......... .......... .......... .......... .......... ..-------- ---------- ----------
#PP993160    .......... .......... A......... .......... .A........ .......... ..C....... .......... .......... .......... .......... .......... ..-------- ---------- ----------
#PP993161    .......... .......... A......... .......... .A........ .......... ..C....... .......... .......... .......... .......... .......... .......--- ---------- ----------
#PP993162    .......... .......... A......... .......... .A........ .......... ..C....... .......... .......... .......... .......... .......... .....----- ---------- ----------
#PP993163    .......... .......... A......... .......... .A........ .......... ---------- ---------- ---------- ---------- ---------- ---------- ---------- ---------- ----------
#PP993164    .......... .......... A......... .......... .A........ .......... ..C....... .......... .--------- ---------- ---------- ---------- ---------- ---------- ----------
#PP993165    .......... .......... A......... .......... .A........ .......... ..C....... .......... .......... ...------- ---------- ---------- ---------- ---------- ----------
#PP993166    .......... .......... A......... .......... .A........ .......... ..C....... .......... .....----- ---------- ---------- ---------- ---------- ---------- ----------
#PP993167    .......... .......... A......... .......... .A........ .......... ..C....... .......... .......... .......... .......... .........- ---------- ---------- ----------
#PP993168    .......... .......... A......... .......... .A........ .......... ..C....... .......... .......... .......... .......... .......... ..-------- ---------- ----------
#PP993169    ........-- ---------- ---------- ---------- ---------- ---------- ---------- ---------- ---------- ---------- ---------- ---------- ---------- ---------- ----------
#PP993170    .......... .......... A......... .....----- ---------- ---------- ---------- ---------- ---------- ---------- ---------- ---------- ---------- ---------- ----------
#PP993171    .......... .......... A......... .......... .A........ .......... A.C....... .--------- ---------- ---------- ---------- ---------- ---------- ---------- ----------
#PP993172    .......... .......... A......... .......... .A........ .......... ..C....... .......... .......... .......... .......... .......... ..-------- ---------- ----------
#PP993173    .......... .......... A......... .......... .A........ .......... ..C....... .......... .......... .......... .......... .....----- ---------- ---------- ----------
#PP993174    .......... .......... A......... .......... .A........ .......... ..C....... .......... ....------ ---------- ---------- ---------- ---------- ---------- ----------
#PP993175    .......... .......... A......... .......... .A........ .......... ..C....... .......... .......... .......... .......... .......... ..-------- ---------- ----------
#PP993176    .......... .......... A......... .......... .A........ .......... ..C....... .......... .......... ..-------- ---------- ---------- ---------- ---------- ----------
#PP993177    .......... .......... A......... .......... .A........ .......... ..C....... .......... .......... .......... .......... .......... ..-------- ---------- ----------
```

B. Deduced amino acid sequence analysis

```
             54         64         74         84         94         104        114        124        134        144
#MT975968    VVAPTRNVTT EKAQRLQIRF YPTQTDDTPN SYRVRYSLNV GDSWVLDMGA TYFDIKGVLD RGPSFKPYGG TAYNPLAPRE AFFNNWVDTE ASKTVITGQM [153]
#KT862805    .......... .......... .......... .......... .......... .......... .......... .......... ......I..G TN........
#KX247375    ---------- ---------- ---------- ---------- ---------- ---------- ------.... .......... .......... ..........
#PP993159    ---------- ---------- ---------- --........ .......... .......... .......... .......... ......I..G TN........
#PP993160    ------.... .......... .......... .......... .......... .......... .......... .......... ......I..G TN........
#PP993161    --------.. .......... .......... .......... .......... .......... .......... .......... ......I..G TN........
#PP993162    ---------- ---------- ---------- --........ .......... .......... .......... .......... ......I..G TN........
#PP993163    -------... .......... .......... .......... .......... .......... .......... .......... ......I..G TN........
#PP993164    ---------. .......... .......... .......... .......... .......... .......... .......... ......I..G TN........
#PP993165    ---------- ---------- ----...... G......... .......... .......... .......... .......... ......I..G TN........
#PP993166    --------.. .......... .......... .......... .......... .......... .......... .......... ......I..G TN........
#PP993167    ---------. .......... .......... .......... .......... .......... .......... .......... ......I..G TN........
#PP993168    ---------. .......... .......... .......... .......... .......... .......... .......... ......I..G TN........
#PP993169    ---------. .......... .......... .......... .......... .......... .......... .......... ......I..G TN........
#PP993170    ---------. .......... .......... .......... .......... .......... .......... .......... ......I..G TN........
#PP993171    ---------- ---....... .......... .......... .......... .......... .......... .......... ......I..G TN........
#PP993172    --------S. .......... .......... .......... .......... .......... .......... .......... ......I..G TN........
#PP993173    ---------- --........ .......... .......... .......... .......... .......... .......... ......I..G TN........
#PP993174    ---------- --........ .......... .......... .......... .......... .......... .......... ......I..G TN........
#PP993175    -------... .......... .......... .......... .......... .......... .......... .......... ......I..G TN........
#PP993176    ---------- --........ .......... .......... .......... .......... .......... .......... ......I..G TN........
```

```
#PP993177    ---------- --........ .......... .......... .......... .......... .......... .......... ......I..G TN........

             154        164        174        184        194        204        214        224        234        244
#MT975968    TTPYENVQGA KDKTAAIVAA LSGVYPDPNI GTAISEMGAL DATSAAQVGL AARFAKVSSD NTRLAYGAYV KPLKNDGSQS INPTPYWVMV SNATNYLGVM  [253]
#KT862805    ......DRS. .......... .......... .P........ .E...D.... .......... .......... .......... .........D .S.AE.....
#KX247375    .......... .......... .......... .......... N......... .......... .......... .......... .........D ..........
#PP993159    ........S. .......... .......... .......... NQ...E.... .......... .......... .......... .........D ....E.....
#PP993160    ........S. .......... .......... .......... NQ...E.... .......... .......... .......... .........D ....E.....
#PP993161    ........S. .......... .......... .......... NQ...E.... .......... .......... .......... .........D ....E.....
#PP993162    ........S. .......... .......... .......... NQ...E.... .......... .......... .......... .........D ....E.....
#PP993163    ........S. .......... .......... .......... .Q...E.... .......... .......... .......... .........D ....E.....
#PP993164    ........S. .......... .......... .......... .Q...E.... .......... .......... .......... .........D ....E.....
#PP993165    ........S. ..T....... .......... .......... .Q...E.... .......... .......... .......... .........D ....E.....
#PP993166    ........S. .......... .......... .......... NQ...E.... .......... .......... .......... .........D ....E.....
#PP993167    ........S. .......... .......... .......... .Q...E.... .......... .......... .......... .........D ....E.....
#PP993168    ........S. .......... .......... .......... .Q...E.... .......... .......... .......... .........D ....E.....
#PP993169    ........S. .......... .......... .......... NQ...E.... .......... .......... .......... .........D ....E.....
#PP993170    ........S. .......... .......... .......... NQ...E.... .......... .......... .......... .........D ....E.....
#PP993171    ........S. .......... .......... .......... NQ...E.... .......... .......... .......... .........D ....E.....
#PP993172    ........S. .......... .......... .......... NQ...E.... .......... .......... .......... .........D ....E.....
#PP993173    ........S. .......... .......... .......... NQ...E.... .......... .......... .......... .........D ....E.....
#PP993174    ........S. .......... .......... .......... NQ...E.... .......... .......... .......... .........D ....E.....
#PP993175    ........S. .......... .......... .......... NQ...E.... .......... .......... .......... .........D ....E.....
#PP993176    ........S. .......... .......... .......... NQ...E.... .......... .......... .......... .........D ....E.....
#PP993177    ........S. .......... .......... .......... .Q...E.... .......... .......... .......... .........D ....E.....

             254        264        274        284        294        304        314        324        334        344
#MT975968    GVEDFSASLT YPDTLLIPPP TEYSEVNRPV MKANRPNYIG FRDNFINLLY HDTGVCSGTL NSERSGMNVV VELQDRNTEL SYQYMLADMM SRHHYYALWN  [353]
#KT862805    .......... .......... .......TG. .......... .......... .......... .......... .......... .......... ..........
#KX247375    .......... .......... .......TG. .......... .......... .......... .......... .......... .......... ..........
#PP993159    .......... .......... .......TG. .......... .......... .......... .......... .......... .......... ----------
#PP993160    .......... .......... .......TG. .......... .......... .......... .......... .......... .......... ----------
#PP993161    .......... .......... .......TG. .......... .......... .......... .......... .......... .......... ..--------
#PP993162    .......... .......... .......TG. .......... .......... .......... .......... .......... .......... .---------
#PP993163    .......... .......... .......TG. .......... .......... .......... .......... ---------- ---------- ----------
#PP993164    .......... .......... .......TG. .......... .......... .......... .......... .......--- ---------- ----------
#PP993165    .......... .......... .......TG. .......... .......... .......... .......... .......... .--------- ----------
#PP993166    .......... .......... .......TG. .......... .......... .......... .......... ........-- ---------- ----------
#PP993167    .......... .......... .......TG. .......... .......... .......... .......... .......... .........- ----------
#PP993168    .......... .......... .......TG. .......... .......... .......... .......... .......... .......... ----------
#PP993169    .......... .......... .......TG. .......... .......... ..-------- ---------- ---------- ---------- ----------
#PP993170    .......... .......... .......TG. .......... .......... .......... .--------- ---------- ---------- ----------
#PP993171    .......... .......... .......TG. .......... .......... .......... .......... I..------- ---------- ----------
#PP993172    .......... .......... .......TG. .......... .......... .......... .......... .......... .......... ----------
#PP993173    .......... .......... .......TG. .......... .......... .......... .......... .......... .....----- ----------
```

```
#PP993174    .......... .......... .......TG. .......... .......... .......... .......... ........-- ---------- ----------
#PP993175    .......... .......... .......TG. .......... .......... .......... .......... .......... .......... ----------
#PP993176    .......... .......... .......TG. .......... .......... .......... .......... .......... ---------- ----------
#PP993177    .......... .......... .......TG. .......... .......... .......... .......... .......... .......... ----------
```

Nucleotides in squares indicate nonsilent mutations

HVR1 (N; 49-243, A.A;17-81) **HVR2 (N; 244-291, A.A; 82-97)**

HVR3 (N;337-42, A.A; 113-143) **HVR4 (N; 484-501, A.A; 162-167)**
